# Influencing factors of anti‐SARS‐CoV‐2‐spike‐IgG antibody titers in healthcare workers: A cross‐section study

**DOI:** 10.1002/jmv.28300

**Published:** 2022-11-18

**Authors:** Julia Reusch, Isabell Wagenhäuser, Alexander Gabel, Annika Eggestein, Anna Höhn, Thiên‐Trí Lâm, Anna Frey, Alexandra Schubert‐Unkmeir, Lars Dölken, Stefan Frantz, Oliver Kurzai, Ulrich Vogel, Manuel Krone, Nils Petri

**Affiliations:** ^1^ Infection Control Unit University Hospital Wuerzburg Wuerzburg Germany; ^2^ Department of Internal Medicine I University Hospital Wuerzburg Wuerzburg Germany; ^3^ Institute for Hygiene and Microbiology University of Wuerzburg Wuerzburg Germany; ^4^ Institute for Virology and Immunobiology University of Wuerzburg Wuerzburg Germany; ^5^ Leibniz Institute for Natural Product Research and Infection Biology – Hans‐Knoell‐Institute Jena Germany

**Keywords:** anti‐SARS‐CoV‐2‐spike IgG, COVID‐19 vaccination, healthcare workers, SARS‐CoV‐2 infection, seroprevalence

## Abstract

Against the background of the current COVID‐19 infection dynamics with its rapid spread of SARS‐CoV‐2 variants of concern (VOC), the immunity and the vaccine prevention of healthcare workers (HCWs) against SARS‐CoV‐2 continues to be of high importance. This observational cross‐section study assesses factors influencing the level of anti‐SARS‐CoV‐2‐spike IgG after SARS‐CoV‐2 infection or vaccination. One thousand seven hundred and fifty HCWs were recruited meeting the following inclusion criteria: age ≥18 years, PCR‐confirmed SARS‐CoV‐2 infection convalescence and/or at least one dose of COVID‐19 vaccination. anti‐SARS‐CoV‐2‐spike IgG titers were determined by SERION ELISA *agile* SARS‐CoV‐2 IgG. Mean anti‐SARS‐CoV‐2‐spike IgG levels increased significantly by number of COVID‐19 vaccinations (92.2 BAU/ml for single, 140.9 BAU/ml for twice and 1144.3 BAU/ml for threefold vaccination). Hybrid COVID‐19 immunized respondents (after infection and vaccination) had significantly higher antibody titers compared with convalescent only HCWs. Anti‐SARS‐CoV‐2‐spike IgG titers declined significantly with time after the second vaccination. Smoking and high age were associated with lower titers. Both recovered and vaccinated HCWs presented a predominantly good humoral immune response. Smoking and higher age limited the humoral SARS‐CoV‐2 immunity, adding to the risk of severe infections within this already health impaired collective.

## 1 INTRODUCTION

Against the background of the ongoing COVID‐19 pandemic^1^ and the current infection dynamics with the rapid spread of SARS‐CoV‐2 variant of concern (VOC) as well as high incidence levels,^2^, ^3^ the immunity of healthcare workers (HCWs) against SARS‐CoV‐2 continues to play a critical role in preventing disease‐related staff shortages and keeping up public health care capacities.^4^, ^5^, ^6^ COVID‐19 vaccines have evolved as a key prevention strategy to reduce the severity of disease and combat the global spread of SARS‐CoV‐2.^7^

The humoral immune response against SARS‐CoV‐2 is investigated to provide forecasts regarding immunity and protection against severe courses of disease.^8^ The low number of studies published to date show a significant correlation between neutralizing antibody titers and prevention from symptomatic SARS‐CoV‐2 infections.^9^ The available data is still insufficient to make any concrete statements on the influencing factors of antibody titers considering the large number of possible combinations of COVID‐19 vaccinations and/or SARS‐CoV‐2 infection.

Previously published studies on humoral anti‐SARS‐CoV‐2‐spike antibodies have been conducted predominantly in small cohorts or for only short observation periods without consideration of demographic factors, quality of life as well as ability to work, particularly in HCWs.^10^, ^11^, ^12^, ^13^

This study examines the seroprevalence of anti‐SARS‐CoV‐2‐spike IgG following SARS‐CoV‐2 infection and/or COVID‐19 vaccination in HCWs and determines factors influencing antibody titers as a cross‐section study.

## 2 METHODS

### 2.1 Study setting

The data presented is part of the prospective CoVacSer cohort study, which examines SARS‐CoV‐2 immunity derived from serial blood samples as well as survey‐based quality of life and ability to work in HCWs after COVID‐19 vaccination and/or SARS‐CoV‐2 infection. HCW were recruited via intranet messages at a tertiary‐care hospital in Germany with approximately 8000 employees. HCW from other institutions were recruited by word‐of‐mouth recommendation.

The CoVacSer study participants had to meet the following inclusion criteria: (i) age ≥18 years, (ii) signed consent form, (iii) 14 days minimal interval after first polymerase chain reaction (PCR) derived confirmation of SARS‐CoV‐2 infection and/or at least one dose of COVID‐19 vaccination independent of the vaccination concept, and (iv) employment in the healthcare sector.

Serum blood samples for anti‐SARS‐CoV‐2‐spike IgG determination were collected combined with pseudonymized CoVacSer study surveys including demographic data, physical condition, and personal risk factors in addition to World Health Organization Quality of Life (WHOQOL‐BREF)^14^, ^15^ and Work Ability Index (WAI) questionnaire.^16^

Only serum blood samples that were accompanied by a signed consent form as well as a fully completed digital questionnaire have been taken into account for the data analysis. Following pseudonymization, the matching of blood sample and survey was mediated based on date of birth and dates of SARS‐CoV‐2 infection or COVID‐19 vaccination.

Participants with vaccines that were not authorized by the European Medicines Agency (EMA) were excluded from this study. The following vaccines have been included due to EMA authorization throughout the data collection period: (i) BNT162b2mRNA (Comirnaty, BioNTech/Pfizer, Mainz/Germany, New York/USA), (ii) mRNA‐1273 (Spikevax, Moderna, Cambridge/USA), (iii) ChAdOx1‐S (VaxZevria, AstraZeneca, Cambridge/GB), (iv) Ad26.COV2‐S (COVID‐19 vaccine Janssen, New Brunswick/USA).^17^

The data presented in this study describes the cross‐sectional seroprevalence of anti‐SARS‐CoV‐2‐spike IgG titers among HCWs after COVID‐19 vaccination and/or SARS‐CoV‐2 infection at the time of study inclusion.

### 2.2 Data collection

The data collection period ranged from the 29th of September 2021 to the 12th of November 2021 during the fourth wave of the COVID‐19 pandemic in Germany^3^ including predominantly wild‐type SARS‐CoV‐2 infections as well as Alpha and Delta VOC.^18^ The federal vaccination campaign in Germany started on 27th December 2020 with the consequent expansion of vaccination capacities.^19^ Due to the initial vaccine shortage, vaccination was carried out according to a tiered plan, with HCWs assigned to the highest priority level.^20^

Mainly HCWs from a single tertiary care hospital participated in the study, but HCWs from surrounding hospitals and private practice were also included in this study.

The questionnaire survey including WHOQOL‐BREF^14^, ^15^ and WAI^16^ was performed using REDCap (Research Electronic Data Capture, projectredcap.org). Questionnaire data were merged with serological data using Excel 2016 (Microsoft Corporation).

### 2.3 SARS‐CoV‐2 IgG ELISA

Anti‐SARS‐CoV‐2‐spike IgG titers were determined by SERION ELISA *agile* SARS‐CoV‐2 IgG (SERION Diagnostics, Wuerzburg, Germany), technically carried out as an enzyme linked immunoassay (ELISA).

The extinction values were photometrically measured operating with the Dynex Opsys MR™ Microplate Reader and Relevation Quick Link (Dynex technologies) at 405 nm wavelength. The extinction was transferred to the manufacturer specific Serion IgG units per ml (U/ml) using the software easyANALYSE (SERION Diagnostics). These units were converted into the internationally established unit Binding Antibody Units per ml (BAU/ml) using the factor 2.1 according to the manufacturer's instructions.^21^

The threshold IgG values in the selected SERION assay were defined as <10.0 U/ml (21.0 BAU/ml) for negative, ≥10.0 U/ml (21.0 BAU/ml) to <15.0 U/ml (31.5 BAU/ml) for results at the borderline and ≥15.0 U/ml (31.5 BAU/ml) as positive. These values were chosen according to manufacturer's instruction and IgG values above the threshold of 31.5 BAU/ml indicate at least a moderate neutralization capacity.^22^ For detecting anti‐SARS‐CoV‐2‐spike IgG antibody levels beyond the maximum limit of 250 U/ml (525.0 BAU/ml), serum blood samples were diluted based on a dilution series with dilution factors both 10 and 100. Consequently, the measurement range of SERION ELISA *agile* SARS‐CoV‐2 IgG could be expanded.

### 2.4 Ethical approval

The study protocol was approved by the Ethics committee of the University of Wuerzburg in accordance with the Declaration of Helsinki (file no. 79/21).

### 2.5 Statistics

The statistical analyses were performed with the statistical programming language R (version 3.1.2).^23^

Statistical differences between the age distributions of male and female HCWs were separately calculated with the Kolmogorov–Smirnov test against the corresponding age distribution within the German population in 2019.^24^

The analysis of anti‐SARS‐CoV‐2‐spike IgG titers was performed on logarithmized IgG titers (Supporting Information: Supplementary Figure 2).

To analyse the effect of physical conditions and personal risk factors on logarithmized anti‐SARS‐CoV‐2‐spike IgG titers, a lasso regression was performed to identify factors that are associated with IgG (Supporting Information: Supplementary Figure 4). This regression model included the factors age, gender, BMI, smoking, immune deficiency, drug intake, time between serum sampling and last SARS‐CoV‐2 immunizing event (infection or vaccination), vaccination concept and other lifestyle parameters obtained from the study questionnaire. Using a tenfold cross‐validation procedure, the model parameters of the lasso regression model were estimated and the model having the lowest mean squared error (MSE) of ~0.62 was chosen (Supporting Information: Supplementary Figure 5). The factors age, gender, BMI, profession, immune deficiency, smoking, satisfaction with the own health status, medical treatment needs, having a meaningful life, concept of vaccination, and time until serum sampling showed associations to the corresponding anti‐SARS‐CoV‐2‐spike IgG titers.

Modeling the differences of IgG titers with respect to the applied vaccination concept (Figure 3), age, gender, BMI, smoking, immune deficiency, time between serum sampling and last SARS‐CoV‐2 immunizing event and further associated factors (Supporting Information: Supplementary Figure 7) defined by the lasso regression, a multiple linear regression model was applied to the data. Estimating the coefficients of this model based on a data set with unequal sample sizes, a generalized least square fit was performed with the R package *nlme*.^25^, ^26^

Based on the estimated coefficients from the regression model, statistical differences between subgroups of the categorical variables (Figure 3, Supporting Information: Supplementary Figure 5) were calculated using the marginal estimated means. The statistically significance of the pairwise differences were calculated with the Tukey statistics. The post hoc pairwise comparisons were performed by using the *emmeans* package.^27^ To correct against multiple testing, the resulting p values were adjusted using the Benjamini‐Yekutielie procedure.^28^ Adjusted *p*‐values below a significance level of 0.05 were considered statistically significant.

The reproducible script of all statistical analyses can be accessed at https://github.com/AlexGa/Influencing-factors-of-Anti-SARS-CoV-2-Spike-IgG-antibody-titres-in-healthcare-workers.

## 3 RESULTS

### 3.1 Specimen collection and participant recruitment

**Figure 1:**
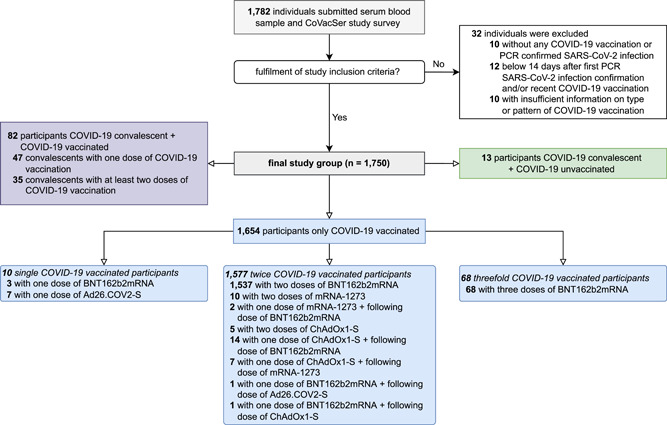
Enrollment of study participants and subjects' characteristics concerning possible combinations of SARS‐CoV‐2 immunization. PCR, polymerase chain reaction.

From the 29th of September 2021 to the 12th of November 2021, 1782 study participants were recruited, who submitted a serum blood sample and completed the CoVacSer study survey. Out of 1782 persons, 1750 (98.2%) participants were finally included. Thirty‐two persons did not meet the inclusion criteria: 10 neither had a PCR‐confirmed SARS‐CoV‐2 infection nor received at least one dose of a COVID‐19 vaccine. Twelve submitted a blood sample before the defined minimal interval of 14 days to the PCR confirmation of the latest SARS‐CoV‐2 infection and/or the recent administration of a COVID‐19 vaccination dose. Ten persons provided insufficient information on type or pattern of COVID‐19 vaccination in the CoVacSer study survey (Figure 1).

Out of the final study group, 13 (0.7%) participants were SARS‐CoV‐2 infection convalescent but not vaccinated against COVID‐19, 82 (4.7%) had a hybrid SARS‐CoV‐2 immunity (SARS‐CoV‐2 infection convalescents with at least one COVID‐19 vaccination). 1577 (90.1%) were twice COVID‐19 vaccinated, 68 (3.9%) threefold vaccinated. Among the twice COVID‐19 vaccinated participants, two consequent doses of BNT162b2mRNA were administered in 1537 cases (87.8%), 10 (0.6%) respondents were vaccinated twice with mRNA‐1273 and 5 (0.3%) have received two doses of ChAdOx1‐S. 14 (0.8%) participants were vaccinated with a first dose of ChAdOx1‐S followed by a second dose of BNT162b2mRNA, 7 (0.4%) followed by a second dose of mRNA‐1273. 2 (0.1%) respondents were COVID‐19 vaccinated with one dose of mRNA‐1273 followed by one dose of BNT162b2mRNA, 1 (0.1%) respondent each received a dose of BNT162b2mRNA followed by a dose of ChAdOx1‐S and Ad26.COV2‐S, respectively. 7 (0.4%) study participants received a single dose of Ad26. COV2‐S.

The median interval between the first and the second dose of BNT162b2mRNA was 21 days (IQR: 21–21), in case of double mRNA‐1273 administration 38 days (IQR: 28–42) and in case of twice ChAdOx1‐S 77 days (IQR: 66–91). For heterologous vaccination schedules with one dose of ChAdOx1‐S followed by one dose of BNT162b2mRNA, the median vaccination interval was 77 days (IQR: 70–82), in case of ChAdOx1‐S followed by mRNA‐1273 84 days (IQR: 80–84).

### 3.2 Study population

1410 out of the 1750 participants assigned themselves to the female gender (80.6%), 340 to the male gender (19.4%). The age of enrolled study participants ranged from 18 to 75 years (median: 39, IQR 30–52), median age of the female participants was 40 years (IQR: 29–53) and 38 years in male participants (IQR: 31 49, Supporting Information: Supplementary Figure 1). In total, 626 (35.8%) of the participants worked as nurses, of which 541 (30.9% of all) were female and 85 (4.9%) were male. Three hundred and twenty‐two (18.4%) were physicians, of which 190 (10.9%) were female and 132 (7.5%) were male. Further professional groups involved other HCWs with contact to patients (22.2% in total) and HCWs without patient contact (23.7% in total).

### 3.3 Anti‐SARS‐CoV‐2‐spike IgG titers depending on vaccination and infection

The collected serum specimens from the enrolled HCWs contained an anti‐SARS‐CoV‐2‐spike IgG range from 6.3 to 6517.8 BAU/ml (geometric mean: 161.4 BAU/ml, IQR: 201.6 BAU/ml). Among participants administered with one dose of the COVID‐19 vaccines, anti‐SARS‐CoV‐2‐spike IgG titers ranged from 15.2 to 402.1 BAU/ml (geometric mean: 92.2 BAU/ml, IQR: 69.8 BAU/ml), with 80.0% (8/10) reaching the positive IgG threshold of 31.5 BAU/ml. In case of double COVID‐19 vaccination, anti‐SARS‐CoV‐2‐spike IgG levels between <6.3 and 4794.7 BAU/ml (geometric mean: 140.9 BAU/ml, IQR: 173.5 BAU/ml) were obtained, with 95.2% (1502/1573) exceeding 31.5 BAU/ml. All study participants that received three or more doses of COVID‐19 vaccination had titers beyond the threshold (68/68, range 79.5–6227.8 BAU/ml, geometric mean: 1114.4 BAU/ml, IQR: 1312.2 BAU/ml).

**Figure 2:**
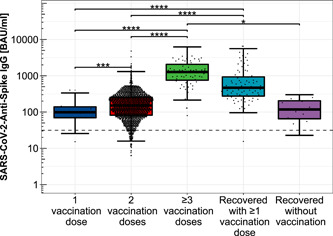
Distribution of anti‐SARS‐CoV‐2‐spike IgG levels depending on immunization scheme. Distribution of anti‐SARS‐CoV‐2‐spike IgG titers among single, double, and threefold COVID‐19 vaccinated participants, only COVID‐19 convalescent study participants as well as hybrid immunized participants including SARS‐CoV‐2 infection convalescence and COVID‐19 vaccination, logarithmically scaled. ****p < 0.0001; ***p < 0.001; **p < 0.01; *p < 0.05. BAU/ml, binding antibody units per milliliter.

In SARS‐CoV‐2 recovered and COVID‐19 unvaccinated participants, the obtained anti‐SARS‐CoV‐2‐spike IgG levels ranged from 22.8 to 299.8 BAU/ml (geometric mean: 105.8 BAU/ml, IQR: 127.7 BAU/ml), 92.3% (12/13) showed IgG levels above 31.5 BAU/ml. Among the hybrid COVID‐19 immunized respondents, the IgG levels between 15.5 and 6517.8 BAU/ml (geometric mean: 525.4 BAU/ml, IQR: 656.9 BAU/ml) were detected, with 98.8% (81/82) above 31.5 BAU/ml (Figure 2, Supporting Information: Supplementary Table 1).

The pairwise differences in SARS‐CoV‐2‐Anti‐Spike IgG titers with respect to different vaccination concepts were statistically significant when comparing one with two administered doses of COVID‐19 vaccination (*p* < 0.001), dual versus at least threefold vaccination (*p* < 0.0001), convalescents without any COVID‐19 vaccination compared to threefold or more vaccinated participants (*p* < 0.05), and comparing the hybrid immunized with both single and double COVID‐19 vaccinated participants (*p* < 0.0001).

### 3.4 Course of time of SARS‐CoV‐2 anti‐Spike IgG levels after last immunizing event

The time interval from the last SARS‐CoV‐2 immunizing event to the moment of study participation ranged from 14 to 569 days (geometric mean: 197.6 days, IQR: 77 days). Among the convalescent study participants, 168–569 days (geometric mean: 267 days, IQR: 96 days) passed since the first PCR confirmation of the latest SARS‐CoV‐2 infection. With respect to statistical outliers, one HCW with a time interval of 569 days between SARS‐CoV‐2 infection and study participation and without additional immunization was removed from further statistical analysis (Supporting Information: Supplementary Figure 4). Within the hybrid COVID‐19 immunized subgroup, the time since last event ranged from 14 to 292 days (geometric mean: 115 days, IQR: 114 days). In case of one administered dose of COVID‐19 vaccination, 14–211 days had passed (geometric mean: 59 days, IQR: 73 days), among the double COVID‐19 vaccinated participants 21–304 days (geometric mean: 221 days, IQR: 66 days), and 14–127 days (geometric mean: 33 days, IQR: 30 days) in case of a threefold COVID‐19 vaccination.

**Figure 3:**
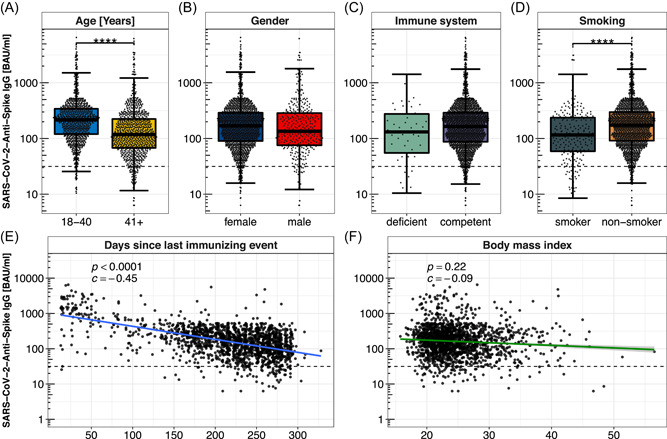
Anti‐SARS‐CoV‐2‐spike IgG levels depending on individual physical properties. (A) Comparison of anti‐SARS‐CoV‐2‐spike IgG levels depending on age in the categories 18–40 years versus older than 40 years. (B) Anti‐SARS‐CoV‐2‐spike IgG titers among smokers versus nonsmokers. (C) Anti‐SARS‐CoV‐2‐spike IgG levels of immune deficient compared to immune competent respondents. (D) Anti‐SARS‐CoV‐2‐spike IgG titers depending on sex. (E) Chronological decline of anti‐SARS‐CoV‐2‐spike IgG levels among double vaccinated respondents, logarithmically scaled, each dot represents a study participant. (F) Anti‐SARS‐CoV‐2‐spike IgG titers depending on BMI logarithmically scaled. BAU/ml, binding antibody units per milliliter. ****p < 0.0001.

A strong association between time since last immunizing event and SARS‐CoV‐2‐Spike IgG titers could be detected (Figure 3E). The IgG titers are negatively correlated (*c* = −0.45) and show a significant decrease over time (*p* < 0.0001).

### 3.5 Identification of anti‐SARS‐CoV‐2‐spike IgG titers influencing factors

Based on the lasso regression model, the following variables were selected to be associated with anti‐SARS‐CoV‐2‐spike IgG levels: age group, gender, BMI, field of employment, immune deficiency, smoking, contentedness with personal health, personal dependency of medical treatment, subjective usefulness of life, contact to SARS‐CoV‐2 infected patients, vaccination concept as well as passed time after the last SARS‐CoV‐2 immunizing event.

The following variables were not associated to the anti‐SARS‐CoV‐2‐spike IgG titers: general contact to patients, respondent subjective evaluation as COVID‐19 risk patient, life quality, long‐term medication, subjective feeling of safety as well as subjective enjoyment of life (Supporting Information: Supplementary Figures 5 and 6).

### 3.6 Influence of individual factors on anti‐SARS‐CoV‐2‐spike IgG titers

Anti‐SARS‐CoV‐2‐spike IgG levels were significantly lower among study participants aged 40 years or older (939/1750, 53.7%, mean: 119.8 BAU/ml, IQR: 148.5 BAU/ml), compared to the younger participants (811/1750, 46.3%, mean: 201.2 BAU/ml, IQR: 214.5 BAU/ml (*p* < 0.0001, Figure 3A).

In the male group, the mean anti‐SARS‐CoV‐2‐spike IgG titer was 146.1 BAU/ml (IQR: 206.4 BAU/ml), among female participants 161.1 BAU/ml (IQR: 194.1 BAU/ml) implementing slightly higher antibody levels in the female subgroup (*p* > 0.05, Figure 3B).

2.6% (45/1750) reported an illness accompanied by an immune deficiency with an obtained mean anti‐SARS‐CoV‐2‐spike IgG titre of 120.0 BAU/ml (IQR: 225.5 BAU/ml) in this subgroup. Comparison of immune deficient to immune competent respondents (mean anti‐SARS‐CoV‐2‐spike IgG level: 159.2 BAU/ml, IQR: 195.6 BAU/ml) regarding antibody levels did not show statistically significant differences (*p* > 0.05, Figure 3C).

222 (12.7%) of the enrolled study participants were smokers with an average level of 11.4 pack‐years (py), which is defined as the number of cigarette packages smoked per day multiplied by years of smoking per person. The geometric mean of anti‐SARS‐CoV‐2‐spike IgG levels in case of smoking was 112.8 BAU/ml (IQR: 179.3 BAU/ml), among the nonsmokers as 166.1 BAU/ml (IQR: 203.2 BAU/ml), portraying smoking as a statistically significant restriction of anti‐SARS‐CoV‐2‐spike IgG levels (*p* < 0.0001, Figure 3D).

The median body weight was 68 kg (IQR: 60–80 kg), with median gender‐specific values of 65 kg (IQR: 58–75 kg) in the female and 80 kg (IQR:–90 kg) in the male subgroup. Combined with the reported body size, with a median of 169 cm (IQR: 164–175 cm, median of 168 cm (IQR: 163–172 cm) in the female and 180 cm (IQR: 176–185 cm) in the male subpopulation), an average body‐mass index of 24 kg/m^2^ was calculated (median of 23 kg/m^2^ among the female, 25 kg/m^2^ among the male study participants. Anti‐SARS‐CoV‐2‐spike IgG levels show very weak associations to BMI, spearman correlation of −0.09 (Figure 3F).

## 4 DISCUSSION

Overall, SARS‐CoV‐2 convalescent as well as COVID‐19 vaccinated and hybrid immunized HCWs presented anti‐SARS‐CoV‐2‐spike levels indicating at least a moderate neutralizing capacity.^22^ Mean anti‐SARS‐CoV‐2‐spike IgG titers increased with the number of administered doses of COVID‐19 vaccines (92.2 BAU/ml for single, 140.9 BAU/ml for double and up to 1144.4 BAU/ml after threefold vaccination). Study participants after SARS‐CoV‐2 infection and without a COVID‐19 vaccination had a mean anti‐SARS‐CoV‐2‐spike antibody titre of 105.8 BAU/ml. In comparison, SARS‐CoV‐2 convalescents with at least a single vaccination (hybrid immunized) had a mean titre of 525.4 BAU/ml, which confirms the importance of the COVID‐19 vaccination as an additional contribution to proper humoral protection against SARS‐CoV‐2 after infection.^29^, ^30^, ^31^ The antibody levels in case of a completed basic immunization, comprising two doses of COVID‐19 vaccination, were significantly lower than in the hybrid immunized group. This cross‐section study highlights the relevance of the COVID‐19 vaccination as a prevention measure, especially in the critical cohort of HCWs that are highly exposed to SARS‐CoV‐2.

Anti‐SARS‐CoV‐2‐spike IgG levels were each statistically significantly lower in smokers and older participants than in the respective comparison groups and decreasin. Further investigations are needed to assess the impact of nicotine consumption in more detail, such as the influence of the number of pack‐years on anti‐SARS‐CoV‐2‐spike IgG levels. COVID‐19 vaccine dose adjustment could provide better humoral protection for smokers who are already recognized as people with an increased risk for a severe course of disease.^32^ Further, a trend towards less, but not statistically significant impairment of the humoral immune response to COVID‐19 vaccination and/or SARS‐CoV‐2 infection was observed among participants suffering from immune deficiency. More precise information regarding the extent and type of the immune suppression and its influence on the humoral immunity is necessary to be able to offer specific and individualized COVID‐19 vaccination recommendations for the future. A restricted humoral SARS‐CoV‐2 immune response of the older participants is a further risk factor for a SARS‐CoV‐2 infection and severe course of disease. This underlines the importance of a COVID‐19 vaccination as a prevention strategy, especially in older HCWs.

However, the significance of the factors that have a negative impact on anti‐SARS‐CoV‐2‐spike IgG levels is still unclear, as threshold values for IgG titers that protect against infection or a severe course are still lacking. Comparability with international research is restricted due to data being presented that is not converted to the global standardized anti‐SARS‐CoV‐2‐spike IgG of BAU/ml following the WHO recommendations in a proportion of other studies.^33^

The obtained anti‐SARS‐CoV‐2‐spike IgG titers showed a significant decrease over time after the last SARS‐CoV‐2 immunizing event indicating a declining humoral immune response after the baseline immunization with COVID‐19 vaccines. This examination represents a cross‐section of data at the beginning of a prospective surveillance study of HCWs that analyzes the humoral immune response as well as quality of life and ability to work.

The data presented should be interpreted considering the possible influence of the following limitations. First, the study population consists of 80.5% female HCWs and thereby represents the typical female‐focused gender composition in the public healthcare sector in Germany with a female share of 75.5% among HCWs in 2019.^34^ However, a gender‐differentiated data analysis in this large study population allows the transferability of the described findings to both HCWs and the public. Second, the vaccines administered are heterogeneously distributed in our cohort, with BNT162b2mRNA double administration accounting for the largest proportion by far. The share of anti‐SARS‐CoV‐2‐spike IgG titers after vaccination with other COVID‐19 vaccines than BNT162b2mRNA is consequently limited and needs to be investigated in further studies. Similarly, the intervals between the individual COVID‐19 vaccine administrations vary as recommended vaccination intervals are not adhered to in all cases. This also limits the generalizability but represents a real‐life scenario. Another limitation resides within unknown, not PCR‐confirmed SARS‐CoV‐2 infections, which might lead to higher anti‐SARS‐CoV‐2‐spike IgG levels after vaccination. Only anti‐SARS‐CoV‐2‐spike IgG were serologically obtained, consequently the differentiation of antibody levels being solely a result of COVID‐19 vaccination or consequences of an unknown SARS‐CoV‐2 infection is not possible. However, unknown SARS‐CoV‐2 infections among HCWs might be seen as less frequent in comparison to the general public due to a strict set of measures to reduce and prevent the spread of SARS‐CoV‐2 in healthcare institutions including regularly implemented employee testing strategies, easy access to SARS‐CoV‐2 PCR when symptomatic or after unprotected exposure to an infected patient.^5^ All subject‐related data, except the serological anti‐SARS‐CoV‐2‐spike IgG measurement, was collected by the means of an electronic questionnaire. Further confounding aspects regarding the anti‐SARS‐CoV‐2‐spike IgG seroprevalence, which are not queried in the survey, cannot be denied. This limitation is counteracted by using the standardized questionnaires WHOQOL‐BREF^14^, ^15^ and WAI.^16^ Consequently, the obtained data is influenced by the subjectivity of the study participants. Additionally, objective assessment of COVID‐19 vaccination and infection as well as specification of SARS‐CoV‐2 VOC was not obtained. Because of pseudonymisation and data privacy of the study participants, query of the exact medication including agent and dose of immune suppression was relinquished. This limits the interpretation of the anti‐SARS‐CoV‐2‐spike IgG levels of HCWs with immune deficiency and its consequences on the immune response after COVID‐19 vaccination.

Based on the presented findings, further examinations regarding the temporal course of anti‐SARS‐CoV‐2‐spike IgG levels and the influence of further COVID‐19 vaccine administrations or SARS‐CoV‐2 infections are urgently needed. In addition, research on correlation of titre values and protection against infection and/or a severe course of disease should be intensified.

## 5 CONCLUSION

The humoral immunity against SARS‐CoV‐2 within the examined cohort of HCWs presents as predominantly good among both convalescent and COVID‐19‐vaccinated participants. The significantly higher anti‐SARS‐CoV‐2‐spike IgG levels in the hybrid immunized subgroup compared with convalescent‐only participants highlight the importance of an additional vaccination after convalescence. Further, significantly higher titers of anti‐SARS‐CoV‐2‐spike IgG were observed after the third vaccination compared with only twice vaccinated participants.

The reduced humoral immune response in smokers and older HCWs adds to the already recognized increased risk for a severe course of disease in these groups of individuals. This is especially important among the highly exposed cohort of HCWs.

## AUTHOR CONTRIBUTIONS

All authors had unlimited access to all data. Julia Reusch, Isabell Wagenhäuser, Alexander Gabel, Manuel Krone, and Nils Petri take responsibility for the integrity of the data and the accuracy of the data analysis. *Conception and design*: Thiên‐Trí Lâm, Anna Frey, Alexandra Schubert‐Unkmeir, Lars Dölken, Stefan Frantz, Oliver Kurzai, Ulrich Vogel, Manuel Krone, and Nils Petri. *Anti‐SARS‐CoV‐2‐spike IgG titre determination*: Julia Reusch and Isabell Wagenhäuser. *Trial management*: Julia Reusch, Isabell Wagenhäuser, Annika Eggestein, Anna Wagner, Manuel Krone, and Nils Petri. *Statistical analysis*: Julia Reusch, Isabell Wagenhäuser, Alexander Gabel, Manuel Krone, and Nils Petri. *Obtained funding*: Oliver Kurzai and Ulrich Vogel. *First draft of the manuscript*: Julia Reusch, Isabell Wagenhäuser, Alexander Gabel, Manuel Krone, and Nils Petri. *Reviewing and modifying the manuscript and approving its final version*: Annika Eggestein, Anna Wagner, Thiên‐Trí Lâm, Anna Frey, Alexandra Schubert‐Unkmeir, Lars Dölken, Stefan Frantz, Oliver Kurzai, and Ulrich Vogel.

## CONFLICTS OF INTEREST

Manuel Krone receives honoraria from GSK and Pfizer outside the submitted work. All remaining authors declare no potential conflicts of interest.

## Supporting information

Supporting information.

Supporting information.

Supporting information.

Supporting information.

Supporting information.

Supporting information.

Supporting information.

Supporting information.

Supporting information.

Supporting information.

## Data Availability

The reproducible script of all statistical analyses can be accessed at https://github.com/AlexGa/Influencing-factors-of-Anti-SARS-CoV-2-Spike-IgG-antibody-titres-in-healthcare-workers Additional data that underlies the results reported in this article, after de‐identification (text, tables, figures, and appendices) as well as the study protocol, statistical analysis plan, and analytic code is made available to researchers who provide a methodologically sound proposal to achieve aims in the approved proposal on request to the corresponding author.
