# Supplementary material for: Influencing factors of anti‐SARS‐CoV‐2‐spike‐IgG antibody titers in healthcare workers: A cross‐section study
Source: J Med Virol. 2022 Nov 18;95(1):e28300. doi: 10.1002/jmv.28300 (PMC9877977; doi:10.1002/jmv.28300)
Supplement: Supplementary file 1 — Supporting information. [file JMV-95-0-s010.docx]

**Title**: *Influencing factors of Anti-SARS-CoV-2-Spike-IgG antibody titres in healthcare workers – A cross-section study*

# Supplementary material

**Supplementary Table 1:**

| **state of vaccination and /or infection** | **single COVID-19 vaccinated** | **double COVID-19 vaccinated** | **threefold COVID-19 vaccinated** | **SARS-CoV-2 convalescent, COVID-19 unvaccinated** | **SARS-CoV-2 convalescent, COVID-19 vaccinated** |
| --- | --- | --- | --- | --- | --- |
| **absolute number** | 10 | 1,577 | 68 | 82 | 13 |
| **geometric mean** | 92.23 | 140.93 | 1,144.40 | 525.43 | 105.76 |
| **minimum of Anti-SARS-CoV-2-Spike IgG** | 15.2 | 6.30 | 79.54 | 15.49 | 22.79 |
| **minimum of Anti-SARS-CoV-2-Spike IgG** | 402.12 | 4,794.72 | 6,227.76 | 6,517.77 | 299.78 |
| **IQR Anti-SARS-CoV-2-Spike IgG** | 69.81 | 173.50 | 1,321.16 | 656.94 | 127.72 |
| **percentage share of the total** | 0.57 | 90.11 | 3.89 | 4.69 | 0.74 |
| **number of specimen beyond positive threshold of 31.5 BAU/ml** | 8 | 1,502 | 68 | 81 | 12 |
| **percentage share of specimen beyond positive threshold of 31.5 BAU/ml** | 80.00 | 95.24 | 100.00 | 98.78 | 92.31 |
