# Supplementary material for: Influencing factors of anti‐SARS‐CoV‐2‐spike‐IgG antibody titers in healthcare workers: A cross‐section study
Source: J Med Virol. 2022 Nov 18;95(1):e28300. doi: 10.1002/jmv.28300 (PMC9877977; doi:10.1002/jmv.28300)
Supplement: Supplementary file 2 — Supporting information. [file JMV-95-0-s008.docx]

**Title**: *Influencing factors of Anti-SARS-CoV-2-Spike-IgG antibody titres in healthcare workers – A cross-section study*

# Supplementary material

**Supplementary Figure 1: Characterization of study population compared to the German general public and HCWs in Germany**

*Supplementary Figure 1: Characterization of study population compared to the German general public and HCWs in Germany*

*Comparison of CoVacSer study population to reference populations.* ***1A*** *portrays the enrolled HCW study population (portrayed in gender separated blue and red bars, n = 1,750) in comparison to the demographic composition of the German general public considering gender and age (black broken line, Kolmogorov-Smirnov-Test) as of December 31, 2020 [1].* ***1B*** *compares the age structure in 10-year categories as a percentage of respondents included in the study (blue bars) with the total number of HCW* *s in Germany (red bars) [2].*

*Data Source: German federal office for statistics, German federal health reporting [1-3].*

**Supplementary Figure 2: Gender separated distribution of HCWs’ profession**

***Supplementary Figure 2****: Relative frequencies of HCWs according to their profession and gender. Normalization of frequencies was performed for each gender separately*.

**Supplementary Figure 3: Distribution of Anti-SARS-CoV-2-Spike IgG levels**

***Supplementary Figure 3****: Density histogram of Anti-SARS-CoV-2-Spike IgG concentrations and of logarithmically scaled Anti-SARS-CoV-2-Spike IgG titres. Obtained logarithmized IgG levels of included study participants are nearly normal distributed.*

**Supplementary Figure 4: Temporal course of Anti-SARS-CoV-2-Spike IgG levels in the study subgroups**

***Supplementary Figure 4****: Temporal course of Anti-SARS-CoV-2-Spike IgG levels depending on time since last event for single (blue dots and regression line), double (red dots and regression line) and threefold or more (green dots and regression line) COVID-19 vaccinated as well as SARS-CoV-2 convalescent (purple dots and regression line) and hybrid immunized participants (turquoise dots and regression line).*

**Supplementary Figure 5: Correlation Anti-SARS-CoV-2-Spike-IgG titres and potentially associated factors**

***Supplementary Figure 5****: Heatmap of pairwise spearman correlation coefficients between Anti-SARS-CoV-2-Spike-IgG titres and all potentially influential factors. Colour scale orange to red indicates positive correlations, while colours from bright blue to dark blue indicate negative correlations.*

**Supplementary Figure 6: Cross-validation and factor selection of lasso regression model**

***Supplementary Figure 6****: Lasso regression for detection of factors associated to Anti-SARS-CoV-2-Spike-IgG titres.* ***6A****: Tenfold cross-validation procedure to determine optimal lambda parameter based on minimal mean-squared error.* ***6B****: Illustrating the shrinkage of coefficients (factors) towards zero with increasing lambda values.*

**Supplementary Figure 7: Post hoc analysis of pairwise differences**

***Supplementary Figure 7:*** *Results of post hoc analysis of pairwise comparisons. The abscissa shows the differences of the estimated marginal means while the ordinate shows all pairwise comparisons with statistically significant differences. The points represent the differences of the estimated marginal means, while the whiskers represent the estimated standard errors*.

**Supplementary Figure 8: Comparison of Anti-SARS-CoV-2-Spike-IgG titres between groups of remaining factors**

***Supplementary Figure 8:*** *Pairwise comparisons of Anti-SARS-CoV-2-Spike-IgG titres between subgroups of remaining factors.*

1. *Statistisches Bundesamt (StBA): Bevölkerung: Deutschland, Stichtag, Altersjahre,*

*Nationalität/Geschlecht/Familienstand*. Available from: <https://www-genesis.destatis.de/genesis/online?sequenz=statistikTabellen&selectionname=12411#abreadcrumb> (Accessed 24 April 2022).

2. *Gesundheitsberichterstattung des Bundes: Gesundheitspersonal in 1.000. Gliederungsmerkmale: Jahre, Deutschland, Alter, Beschäftigungsart, Beruf*

Available from: <https://www.gbe-bund.de/gbe/!pkg_olap_tables.prc_set_orientation?p_uid=gast&p_aid=25632989&p_sprache=D&p_help=2&p_indnr=96&p_ansnr=82673282&p_version=2&D.000=1&D.002=1&D.767=1&D.489=2> (Accessed 24 April 2022).

3. *Statistisches Bundesamt (StBA): Health personnel: Germany, years, facilities, sex*. Available from: <https://www-genesis.destatis.de/genesis/online?sequenz=tabelleErgebnis&selectionname=23621-0001&language=en#abreadcrumb> (Accessed 24 May 2022).
