# Supplementary figures and images for: Influencing factors of anti‐SARS‐CoV‐2‐spike‐IgG antibody titers in healthcare workers: A cross‐section study
Source: J Med Virol. 2022 Nov 18;95(1):e28300. doi: 10.1002/jmv.28300 (PMC9877977; doi:10.1002/jmv.28300)

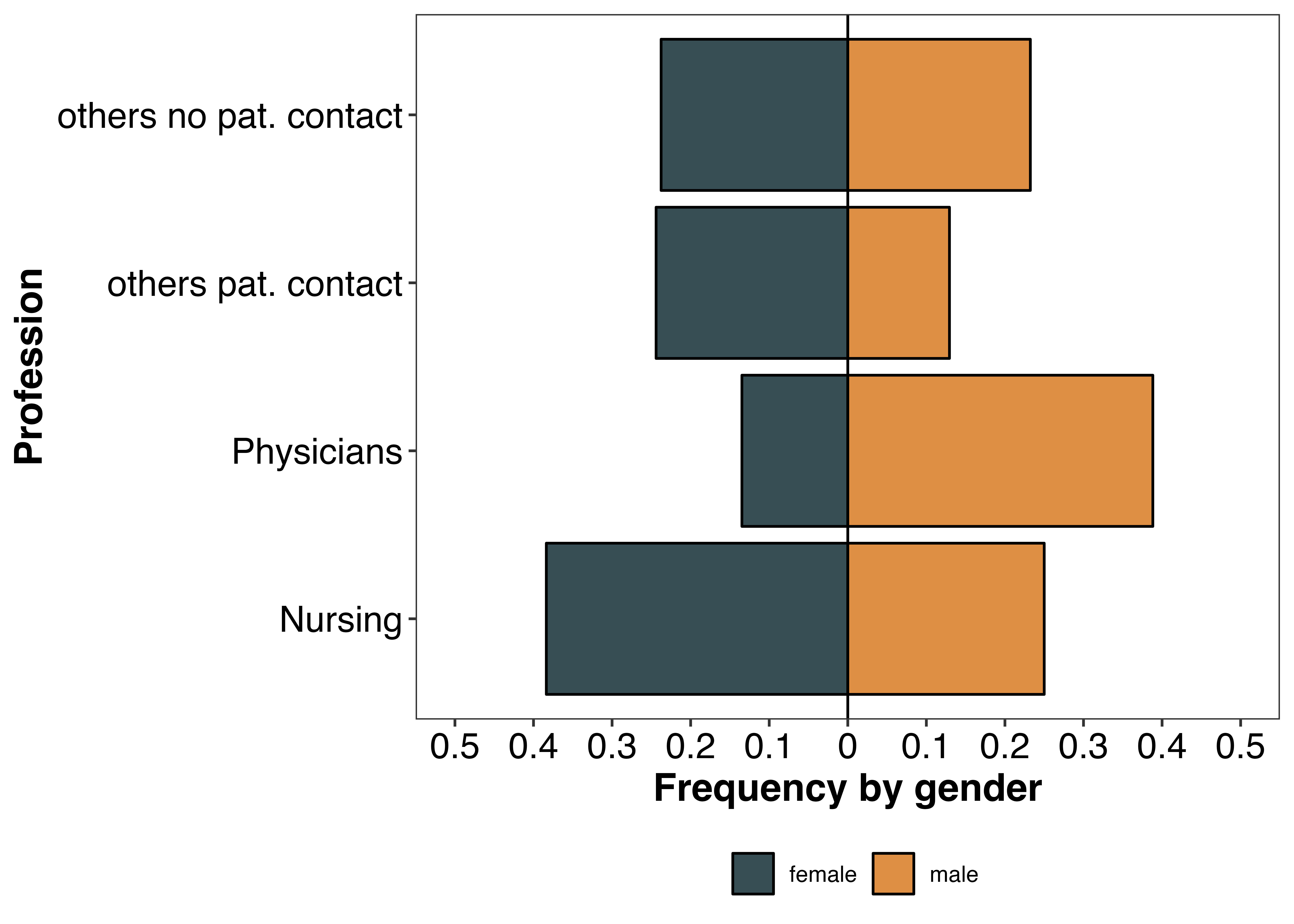

Supplement: Supplementary file 4 — Supporting information. [file JMV-95-0-s003.jpeg]

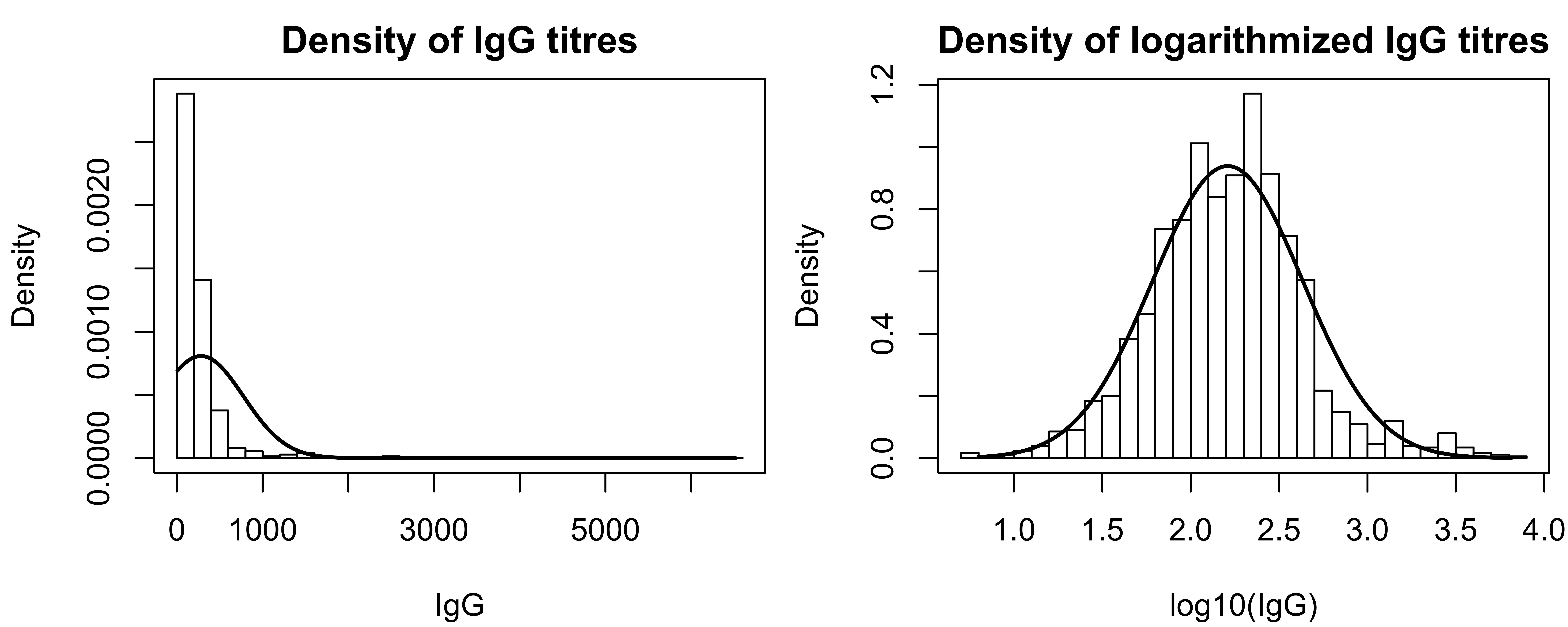

Supplement: Supplementary file 5 — Supporting information. [file JMV-95-0-s005.jpeg]

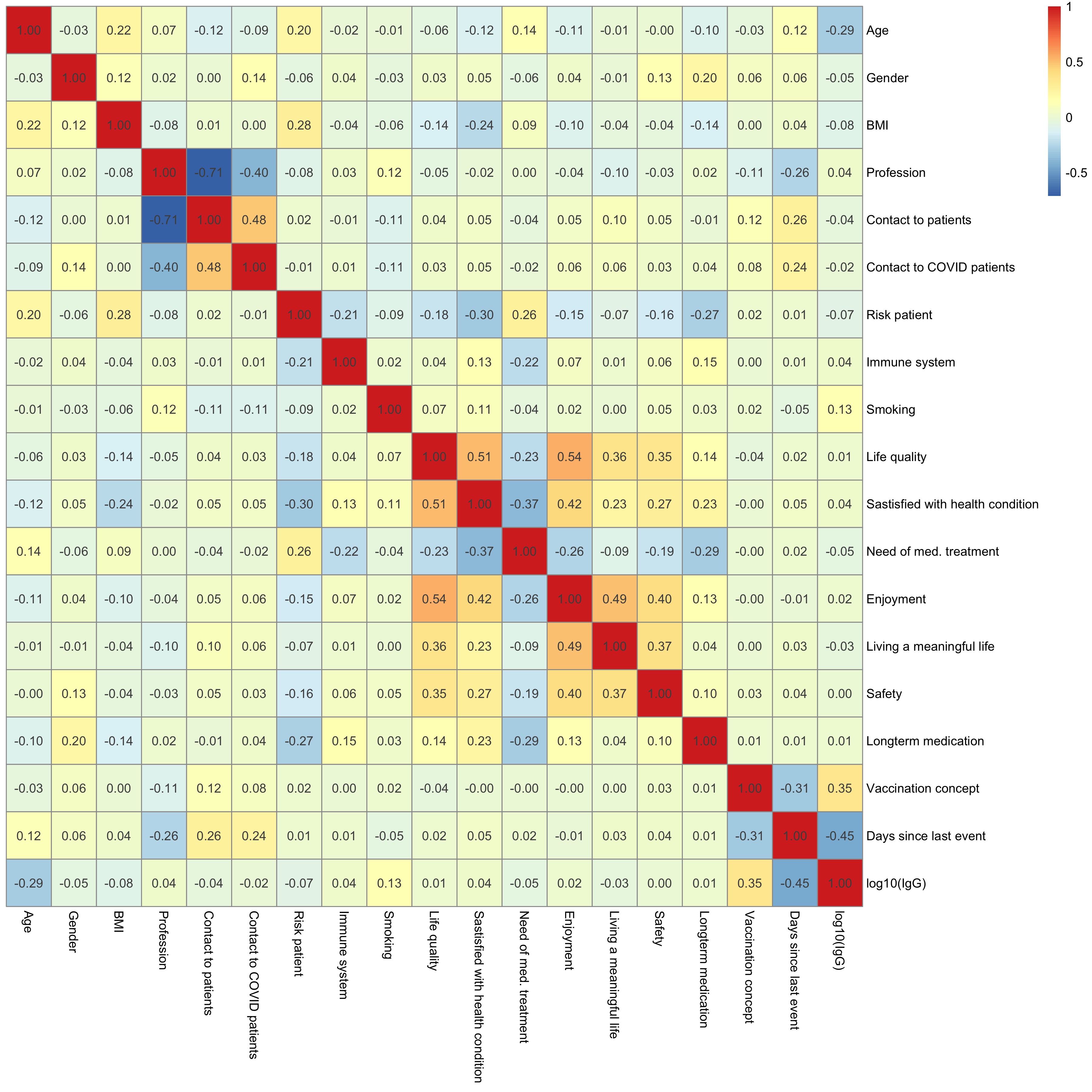

Supplement: Supplementary file 7 — Supporting information. [file JMV-95-0-s009.jpeg]

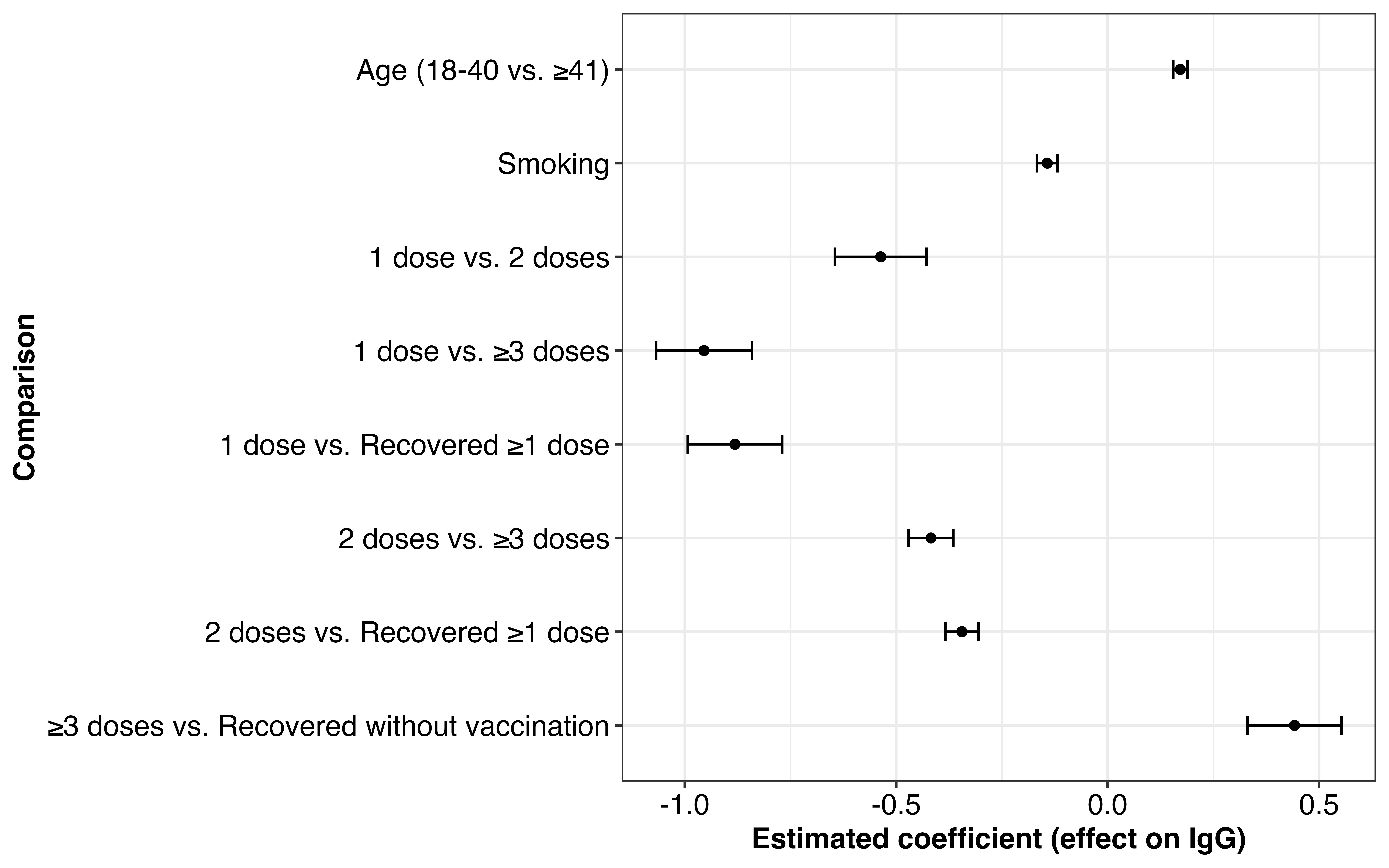

Supplement: Supplementary file 9 — Supporting information. [file JMV-95-0-s002.jpeg]
